# Reference quality genome sequence of Indian pomegranate cv. ‘Bhagawa’ (*Punica granatum* L.)

**DOI:** 10.3389/fpls.2022.947164

**Published:** 2022-09-15

**Authors:** P. Roopa Sowjanya, Parashuram Shilpa, Goudappa Prakash Patil, Dhinesh Karuppannan Babu, Jyotsana Sharma, Vipul R. Sangnure, Dhananjay M. Mundewadikar, Purushothaman Natarajan, Arvind Rajiv Marathe, Umesh K. Reddy, Vikram Nripendra Singh

**Affiliations:** ^1^ICAR-National Research Centre on Pomegranate (NRCP), Solapur, India; ^2^CSIR-National Chemical Laboratory, Pune, India; ^3^Gus R. Douglass Institute and Department of Biology, West Virginia State University, Institute, WV, United States

**Keywords:** pomegranate, PacBio, Long Terminal Repeats Assembly Index (LAI), genome sequencing, hybrid genome assembly

## Abstract

Pomegranate is an important fruit crop for ensuring livelihood and nutrition security in fragile semi-arid regions of the globe having limited irrigation resources. This is a high-value, nutritionally rich, and export-oriented agri-commodity that ensures high returns on investment to growers across the world. Although it is a valuable fruit crop, it has received only a limited genomics research outcome. To fast-track the pomegranate improvement program, *de novo* whole-genome sequencing of the main Indian cultivar ‘Bhagawa’ was initiated by the Indian Council of Agricultural Research–National Research Center on Pomegranate (ICAR–NRCP). We have demonstrated that a combination of commercially available technologies from Illumina, PacBio, 10X Genomics, and BioNano Genomics could be used efficiently for sequencing and reference-grade *de novo* assembly of the pomegranate genome. The research led to a final reference-quality genome assembly for ‘Bhagawa’ of 346.08 Mb in 342 scaffolds and an average N50 of 16.12 Mb and N90 of 1088.62 Kb. This assembly covered more than 98% of the estimated pomegranate genome size, 352.54 Mb. The LTR assembly index (LAI) value of 10 and 93.68% Benchmarking Universal Single-Copy Orthologs (BUSCO) completeness score over the 1,440 ortholog genes of the completed pomegranate genome indicates the quality of the assembled pomegranate genome. Furthermore, 29,435 gene models were discovered with a mean transcript length of 2,954 bp and a mean coding sequence length 1,090 bp. Four transcript data samples of pomegranate tissues were mapped over the assembled ‘Bhagawa’ genome up to 95% significant matches, indicating the high quality of the assembled genome. We have compared the ‘Bhagawa’ genome with the genomes of the pomegranate cultivars ‘Dabenzi’ and ‘Taishanhong.’ We have also performed whole-genome phylogenetic analysis using Computational Analysis of Gene Family Evolution (CAFE) and found that *Eucalyptus grandis* and pomegranate diverged 64 (60–70) million years ago. About 1,573 protein-coding resistance genes identified in the ‘Bhagawa’ genome were classified into 32 domains. In all, 314 copies of miRNA belonging to 26 different families were identified in the ‘Bhagawa’ genome. The reference-quality genome assembly of ‘Bhagawa’ is certainly a significant genomic resource for accelerated pomegranate improvement.

## Introduction

Pomegranate (*Punica granatum* L.), a diploid fruit crop species (2n = 16), is a member of the lythraceae family (Graham and Graham, 2014; Berger et al., 2016). The *Punica* genus has two species: *P. granatum* and *P. protopunica*. All cultivated-type pomegranates belong to *P. granatum*. *P. protopunica* is considered an ancestor of cultivated pomegranates and has contributed to the evolutionary process. However, *P. protopunica* is endemic to the Socotra Islands (Yemen) and is not available in most of the major pomegranate-growing countries of the world. To trace the evolutionary history of cultivated pomegranate, two wild pomegranate types, *Daru* and *P. granatum* var. Nana, were used (Moriguchi et al., 1987; Guarino et al., 1990; Mars, 2000; Levin, 2006; da Silva et al., 2013; Ferrara et al., 2021).

Pomegranate is globally grown on approximately 0.55 million ha with a production of about 6.5 million tonnes, and it is considered an important fruit crop in semi-arid tropical areas (Ferrara et al., 2021). It is an economically important fruit crop with high nutraceutical value. Pomegranate has approximately 11.33 mmol of antioxidants, such as punicalagin and other ellagitannin-based compounds per 100 g of fruit. These antioxidants have medicinal properties for heart disease and prostate cancer (Halvorsen et al., 2002; Holland et al., 2009; Johanningsmeier and Harris, 2011).

Pomegranate cultivation started in 3000 BC in Central Asia (Chandra et al., 2010). The pomegranate crop has versatile adaptability because of its hardy nature and low water requirements across the Mediterranean, tropical and subtropical regions of Iran, India, China, Turkey, Spain, Tunisia, Morocco, and Afghanistan. Globally, India is the largest producer of pomegranate, with 2.8 million tonnes of annual production contributing about 40% of the global share, followed by China (1.6 million tonnes), Iran (0.7 million tonnes), and Turkey (0.5 million tonnes) (Ferrara et al., 2021). Pomegranate is considered a strategic crop for ensuring nutritional and livelihood security in water-scarce regions of the world, hence, to some extent, it can help to mitigate global warming.

Due to of the alluring monetary return per unit area from this crop and increased demand for table and processed products with high export potential, pomegranate cultivation has experienced a tremendous increase in area, production, and export from India during the last 2 decades. In India, the pomegranate acreage is 0.275 million ha, with an annual production of 3.256 million tonnes. It is extensively grown in Maharashtra, Gujarat, Karnataka, Andhra Pradesh, and Telangana states and is quickly being established in Himachal Pradesh, Rajasthan, and Madhya Pradesh. Small areas of pomegranate are under cultivation in Tamil Nadu, Punjab, Haryana, Jharkhand, and Jammu and Kashmir (2019-20^1^). Export of pomegranate has increased from 18.21 thousand MT (Rs. 710 million) in 2010–2011 to about 99.04 thousand MT (Rs.6888 million) in 2021–2022^2^.

Although the area under pomegranate cultivation is increasing because it is grown in low-input and risk-prone marginal environments, there is a large gap between the expected yield potential (average productivity of some of the major pomegranate-producing countries is about 20 tonnes/ha) and the realized yield potential on farmers’ fields (∼12 tonnes/ha) in India. This situation could be due to various biotic and abiotic stresses affecting the crop. Therefore, to accelerate the application of genomics to improve the yield and quality of pomegranate, we assembled the genome sequence of the Indian pomegranate cultivar ‘Bhagawa’ and performed further analysis. The Indian pomegranate industry is ruled by a single cultivar, ‘Bhagawa,’ having excellent exportable fruit qualities with high export demand. It is also preferred for domestic consumption. However, this variety is highly susceptible to major diseases and pests, such as bacterial blight, *Ceratocystis* wilt, fruit sucking moths, etc., that are significantly reducing the yield potential of this variety. Therefore, we selected ‘Bhagawa’ for assembling a quality genome to decode important genes for resistance/susceptibility, which can be later targeted for genome-editing applications to improve this variety.

Despite the availability of the draft genome sequences of cv. ‘Taishanhong’ and ‘Dabenzi’ pomegranate of China and transcriptome assemblies, deeper knowledge of the genetic basis of yield, quality, and stress tolerance for genetic improvement is lacking in pomegranate (Ono et al., 2011; Ophir et al., 2014; Saminathan et al., 2016; Qin et al., 2017; Yuan Z. et al., 2018). Also, the unavailability of a high-quality reference genome limits molecular studies in pomegranate. Therefore, in the present study, we aimed to assemble the high-quality genome of cv. ‘Bhagawa’ by using third- and fourth-generation sequencing technologies to capture the entire genome with high continuity. We also compared the assembly quality of our genome with available draft genomes of pomegranate. The availability of the complete genome sequence will accelerate the use of pomegranate genepool resources in molecular breeding. Also, we developed genome-wide simple sequence repeat (SSR) markers for gene discovery and molecular breeding applications. The genomic resources from the current study will benefit the pomegranate research community in discovering the trait-specific genes. It will also increase the efficiency of pomegranate improvement by integrating novel biotechnological tools, such as genome editing and genomics-assisted selection, to complement conventional breeding programs of pomegranate variety improvement with resistance to biotic and abiotic stresses.

## Results

### Genome sequencing and *de novo* genome assembly

**FIGURE 1:**
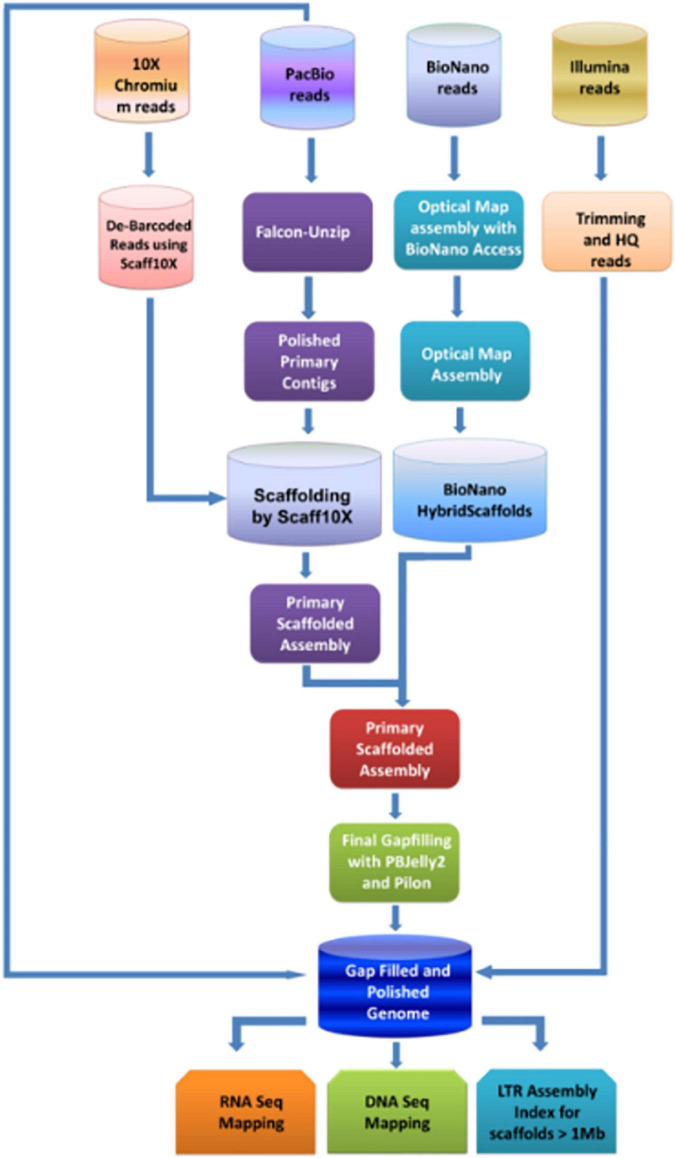
Flow diagram of the strategy used for assembling the genome of pomegranate cv. ‘Bhagawa’.

The study aimed to generate the high-quality reference-level genome of the Indian pomegranate cultivar ‘Bhagawa.’ To achieve this, we opted for short- to long-read sequencing with second- to fourth-generation technologies, such as Illumina, 10X genomics, PacBio, and BioNano Optical mapping. Hence, we could generate enormous genomic data for each technology (Table 1). Using the direct-label and stain (DLS) technology of the Bionano platform, we generated data of 1191.68 Gb covering 340.4 Mb of the genome (∼352.54 Mb of an estimated genome) followed by the use of the HiSeqX platform generating data of 77.08 Gb covering 220 Mb of the genome. The sequence information generated from all these technologies could lead to hybrid scaffolding and the development of a reference-quality genome for ‘Bhagawa.’ The detailed methodology with steps followed to complete the genome is depicted in Figure 1.

**TABLE 1:** Summary of data generated by using genome sequencing platforms for *Punica granatum* cv. ‘Bhagawa’.

DNA preparation protocols	Sequencing/capturing platform	Data generated (Gb)	Genome coverage ∼ 350 Mb
Paired End	Illumina HiSeq 2500	75.46	215.6
10X Chromium	HiSeq X	77.08	220
SMRT-Bell	PacBio Sequel	31.42	89.74
DLS	BioNanoSaphyr	1191.68	340.4

Direct-label and stain — DLS.

**FIGURE 2:**
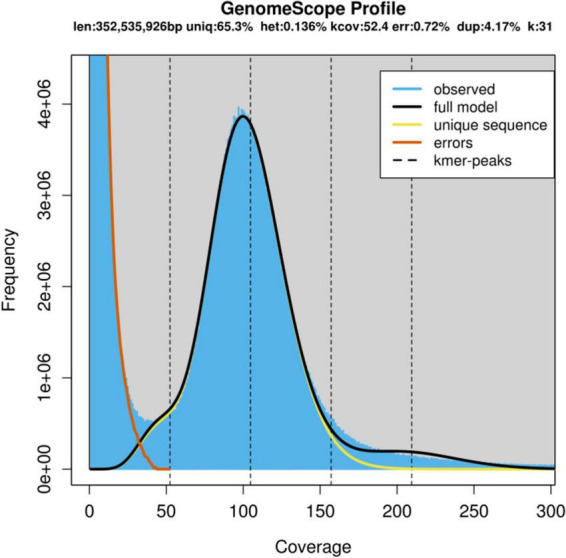
Genome scope profile showing the genome size and heterozygosity rate as estimated by using k-mers from the ‘Bhagawa’ genome.

From the results of preliminary k-mer genome survey analysis for cv. ‘Bhagawa’ using 10X Chromium datasets, the final genome size was estimated at 352,535,926 bp (352.54 Mb, *k* = 31). The heterozygosity rate was 0.14% and the repeat ratio was 4.17%, with 65.3% unique sequences and 0.72% error rate, as depicted in Figure 2.

We assembled the genome by using datasets generated from different sequencing platforms and an array of assembling software shown in Table 2. Results of five approaches combining different sequencing technologies and assembly software were compared. Among the five approaches, the Bio-nano genomics (BNG) and BNG-assisted Super Scaffolds coupled with BioNano Access assembly software yielded higher genome coverage (343.27–353.59 Mb) with limited sequences, 78–374, and a higher average N50 of 16.11–16.31 Mb for the scaffolds. Thus, BNG coupled with BioNano Access was the best to obtain high-quality assembly of genomes with higher contiguity than 10X and PacBio contigs coupled with Super Nova, Falcon-unzip, and Scaff10x assembly (Table 2). Furthermore, we polished the genome assembly to obtain the final assembly by using PBJelly2 and Pilon. The final reference-quality genome was 346.08 Mb with only 342 scaffolds and an average N50 of 16.12 Mb and N90 of 1088.62 Kb (Table 2). This assembly covered 98% of the estimated size of the pomegranate genome, 352.54 Mb.

**TABLE 2:** Statistics at each step of the pomegranate genome assembly.

Technology & assembly	Software	Length (Mbp)	No. of sequence/no. of scaffold	Contig/scaffold N50 (Mbp)	Contig/scaffold N90(Kbp)	Longest contig/scaffold (Mbp)	Median contig/scaffold (Kbp)
10X Scaffolds	SuperNova	331.1	12063	1.31	9.6	7.16	3.6
PacBio Contigs	Falcon-unzip	337.12	446	6.8	487.9	18.95	77.27
PacBio contigs + 10X reads (hybrid scaffolds)	Scaff10x	337.72	432	9.88	508.06	18.95	73.46
BNG-assisted SuperScaffolds	BioNano Access (All Scaffolds)	343.27	374	16.11	1059.69	22.45	54.17
PBJelly2 and Pilon polished genome	PBJelly2 and Pilon	346.08	342	16.12	1088.62	22.45	65.89

**FIGURE 3:**
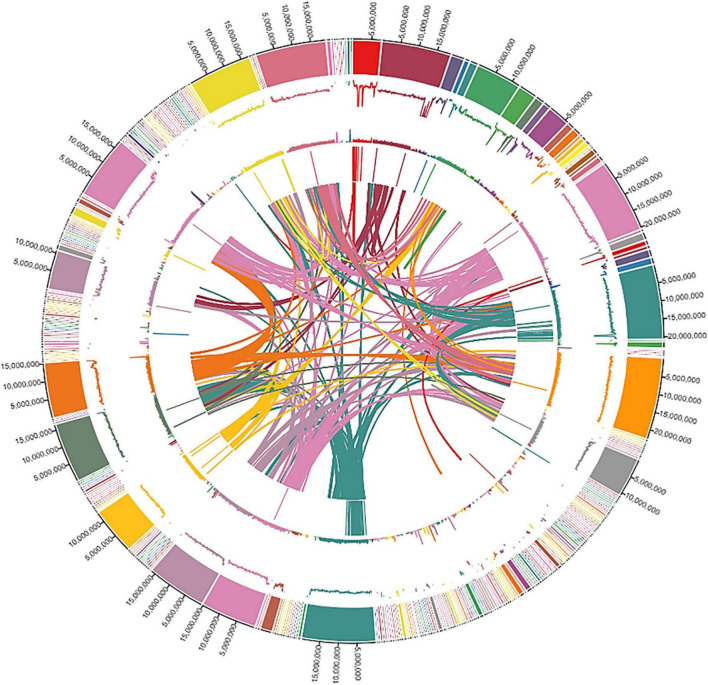
The genomic landscape of Indian pomegranate cv. ‘Bhagawa.’ Shows the distribution of the scaffold length, guanine and cytosine GC content, repeat content, gene density, and interactions of scaffolds, with densities calculated in 100-kb window size. The tracks from outside to inside are (1) circular representations of scaffolds 1-342, (2) distribution of Guanine and Cytosine (GC) content in scaffolds, (3) distribution of repeat content, (4) distribution of gene density in scaffolds, and (5) synteny blocks from linked regions of scaffolds.

We assessed the genome quality by using the LTR Assembly Index (LAI) based on LTR retrotransposons that account for the largest genome component in most plant genomes. Our genome had an LAI index of 10, indicating a reference-quality genome as per the LAI index scale of ≤ 10 to 20 for reference-quality genomes (Ou et al., 2018). Finally, the qualities of different assemblies were assessed by using BUSCO. The complete genomic landscape of the ‘Bhagawa’ genome is presented in Figure 3.

To judge our genome’s quality, we compared the assembly statistics of the ‘Bhagawa’ genome with the other two draft genomes for pomegranates cv. ‘Taishanhong’ and ‘Dabenzi.’ According to the assembly parameters listed, our genome qualifies for all parameters with high standards as compared with the previous genomes (Table 3). For instance, our final assembly covered 346.08 Mb (98% of estimated genome size) in 342 scaffolds with an N50 of 16.12 Mb and a contig N50 of 6.8 Mb with high GC content (41.01%) **(NCBI database Bio project: PRJNA562100, PRJNA505392, PRJNA505397, PRJNA505398, PRJNA505582, and PRJNA445950)**. A total of 29,435 gene models were discovered, with a mean transcript length of 2,954 bp and a mean coding sequence length 1,090 bp as compared with the previous draft genomes (Supplementary File 1). The LAI index that was calculated for all three genomes was 10 for ‘Bhagawa,’ 8 for ‘Taishanhong,’ and 2.5 for ‘Dabenzi,’ so our genome is of high reference quality as compared with the other draft pomegranate genomes. However, more recently, a chromosome-scale genome assembly of soft-seeded pomegranate cv. ‘Tunisia’ was reported; the assembly involved the combined use of single-molecule sequencing and high-throughput chromosome conformation capture techniques. The genome covers 320.31 Mb, with 39.96 Mb scaffold N50 value and 4.49 Mb contig N50 value, and the genome includes 33,594 protein-coding genes (Luo et al., 2020).

**TABLE 3:** Comparative metrics of the ‘Bhagawa’ genome with genomes of other pomegranate varieties available in National Center for Biotechnology Information (NCBI).

S. no.	Assembly parameters	‘Taishanhong’ (illumina)* (Yuan Z. et al., 2018)	‘Dabenzi’ (illumina)* (Qin et al., 2017)	‘Bhagawa’ (hybrid approach)
1	Estimated genome size (Mb)	336	328.13	352.53 (*k* = 31)
2	Total size of assembled scaffolds (Mb)	274	296.38	346.08
3	Number of scaffolds (≥ 1 kb)	2,117	2,601	342
4	N50 scaffold length (Mb)	1.7	2.3	16.12
5	Longest scaffold (Mb)	7.6	9.97	22.45
6	Total size of assembled contigs (Mb)	269	N/A	337.7
7	Number of contigs (≥ 1 kb)	7,088	N/A	446
8	N50 contig length	97 Kb	82.31 Kb	6.8 Mb
9	Largest contig	528.6 Kb	N/A	18.89 Mb
10	GC content (%)	39.2	39.64	41.01
11	Number of gene models**	30,903	29,226	29,435
12	Mean transcript length (bp)**	2332.8	2,543	2,954
13	Mean coding sequence length (bp)**	1110.4	1,077	1,090
14	Mean number of exons per gene**	4.52	4	5.1
15	Mean exon length (bp)**	245.9	308	286

*Based on genomes submitted in NCBI.

**Based on annotation information either in NCBI or in the manuscript.

N/A, not available.

### Synteny between genome assemblies

**FIGURE 4:**
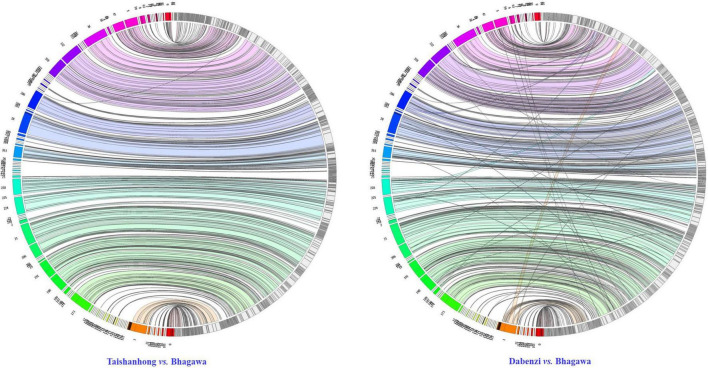
Circos visualization of the syntenic relationship of ‘Bhagawa’ genome with the genomes of ‘Taishanhong’ and ‘Dabenzi’ pomegranate varieties.

To assess the syntenic relationships between the newly assembled ‘Bhagawa’ genome and the two previously reported draft genomes, we performed global whole-genome alignments using Minimap2 with parameter “asm10”; the synteny relationships were visualized as a Jupiter Plot (Jones et al., 2014). A Circos-based genome assembly consistency plot was used to view large-scale translocations and other large structural variations (Krzywinski et al., 2009; Jones et al., 2014). The connecting bands within the circle represent regions of synteny, whereas the blocks on the arc of the circle represent the largest scaffolds in the assembly. The lack of diagonal lines extending from the middle of the scaffold block suggests no definite breaks in synteny between the two assemblies at 10-Kb resolution. We found perfect macro synteny between the ‘Dabenzi’ vs. ‘Bhagawa’ genome compared to ‘Taishanhong’ vs. ‘Bhagawa’ (Figure 4). These results indicated higher syntenic relationships between ‘Bhagawa’ and ‘Dabenzi’ followed by ‘Taishanhiong.’ Several scaffolds had multiple hits in the ‘Taishanhong’ vs. ‘Bhagawa’ plot, thus indicating low levels of genome rearrangements between these two genomes. Similar results were observed with the ‘Dabenzi’ genome plotted against ‘Taishanhiong.’

### Structural variation (SV) analyzes

An SV represents a major source of genetic diversity, we investigated overall SVs in the pomegranate genome. We aligned the ‘Bhagawa’ genome with the ‘Taishanhong’ and ‘Dabenzi’ genomes separately by using Assemblytics (Supplementary Figure 2). Here, we tried to identify SVs for six different classes: insertion–deletion, repeat expansion and contraction, and tandem expansion and contraction, focusing on 1–10 Kb between our ‘Bhagawa’ genome and the other two draft pomegranate genomes. We identified 5.7 Mb and 9.2 Mb SVs for ‘Taishanhong’ and ‘Dabenzi’, respectively, with ‘Bhagawa’ as a reference (Supplementary Tables 1A,B and Supplementary Figure 2). Although, all the three cultivars belong to the same species, *P. granatum*, they have accumulated much higher SVs at the nucleotide level in the genome. The SV graph indicates that except for tandem contraction, much higher SVs were observed between ‘Bhagawa’ and ‘Dabenzi’ than between ‘Bhagawa’ and ‘Taishanhong’ for all the variant classes. Also, the SVs frequency decreased with the increasing size of the nucleotide sequences analyzed for each class. Among the SVs, insertions and deletions contributed much greater to total variations in the genomes, followed by repeat expansion and contraction.

### Repetitive sequences

Most plant genomes have highly repetitive regions with transposable elements. We have analyzed the pomegranate genome for these aspects to reveal the basic structural features of the genome. *De novo* analysis of repetitive elements using the Repeat Modeler software revealed a large proportion of repetitive DNA, comparable to the other higher eukaryotic genomes (Table 4). Repeat Modeler generated 1,765 different families of repeats known as the *de novo* repeat reference library. A total of 496,570 repetitive elements were in the genome, covering 179,092,850 bp of sequence. Most of the repetitive elements were the interspersed type, consisting of Class I (retro transposons) and Class II (DNA transposons) elements followed by unclassified elements, small RNA, and satellites. Simple direct repeats and low complexity repeats represented only 1.03% and 0.18% of the total repetitive elements. Classification of the observed transposable elements into known classes revealed that most repetitive sequences were retrotransposons (18.27%), whereas 1.10% were DNA transposons (Table 4). The most abundant repeats were long-terminal repeat elements (17.33%), of which 13.57% were Gypsy-type elements and 2.95% Copia-type elements (Table 4).

**TABLE 4:** Different types of repeat elements identified in the ‘Bhagawa’ genome.

Repeats categories	Number of elements	Length (bp)	Percentage of genome
Retroelements (Class I):	46,207	632,42,519	18.27
-SINEs	300	91,705	0.03
-LINEs (L1/CIN4)	6,571	316,0098	0.91
-LTR elements:	39,336	59,990,716	17.33
-BEL/Pao	1,548	241,755	0.07
-Ty1/Copia	8,596	10,208,967	2.95
-Gypsy	26,478	46,959,638	13.57
-Retroviral	1,204	862,209	0.25
-Others	1,510	17,181,47	0.50
DNA transposons (Class II):	6,188	38,20,656	1.10
-hobo-Activator	992	580,336	0.17
-Tourist/Harbinger	1,733	10,14,546	0.29
-Rolling-circles	3,371	21,68,970	0.63
-Others	92	56,804	0.02
Unclassified	333,789	106,506,469	30.78
Total interspersed repeats		173,569,644	50.16

### Gene prediction and functional annotation

**FIGURE 5:**
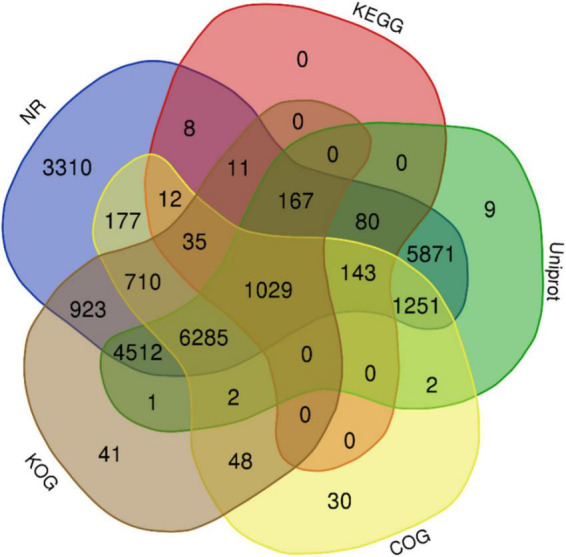
Functional annotation of the predicted genes from ‘Bhagawa’ genome by using NCBI, NR, Kyoto Encyclopedia of Genes and Genomes, Uniport, Gene Ontology, Cluster of Orthologous Groups, and Eukaryotic Orthologous Groups databases.

**FIGURE 6:**
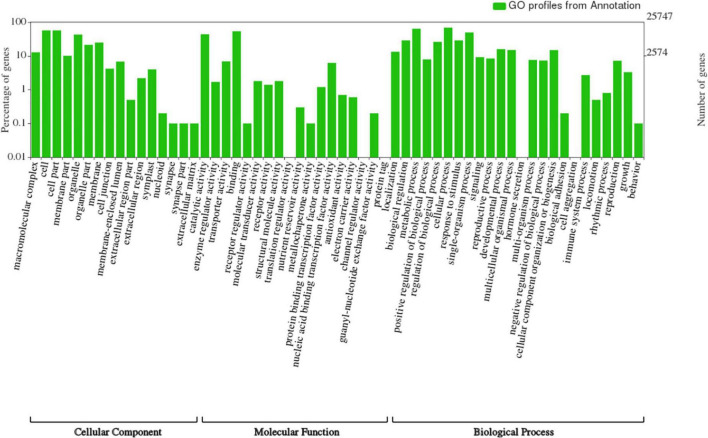
Functional classification of genes from ‘Bhagawa’ genome.

The complete genome sequence of ‘Bhagawa’ (∼346 Mb) was analyzed for gene prediction *ab initio* and by using a homology-based approach with the BRAKER/MAKER pipeline. A total of 29,435 genes were predicted with an average size of 2,954 bp, with average exon and intron sizes of 286 and 368 bp, respectively (Supplementary File 2). All predicted genes were functionally annotated by following a consensus approach of known homologous or predictive sequence signatures by Cluster of Orthologous Groups, Gene Ontology, InterProScan, Kyoto Encyclopedia of Genes and Genomes, Uniprot, and EggNOG (Figures 5, 6). In total, 96.04% of genes had sufficient similarity entries in databases to tentatively assign gene functions. Only 3.96% of genes remain unannotated. The overall GC content of the genome was 41.01% (Table 3). Additionally, 617 transfer RNA genes were predicted in the genome.

The complete genome represented 93.68% of the 1440 ortholog genes, with 64 missing and 27 fragmented genes, according to step-wise BUSCO assessment of the Embryophyta lineage. We also successfully validated the four transcriptome data samples (NCBI database: SRR5187757, SRR5187758, SRR5187763, and SRR5187764) of bacterial blight-challenged pomegranate tissues by mapping them onto the reference genome. As a result, 85% of 95% of reads showed significant matches to the genome (Supplementary Figure 3) (Kozlov et al., 2019). These results indicated an extremely low proportion of misassemblies in the gene-rich regions. The transcriptome assembly does not necessarily represent all pomegranate genes; it is restricted to the tissue types used, and genes expressed at low levels are likely under-represented.

**FIGURE 7:**
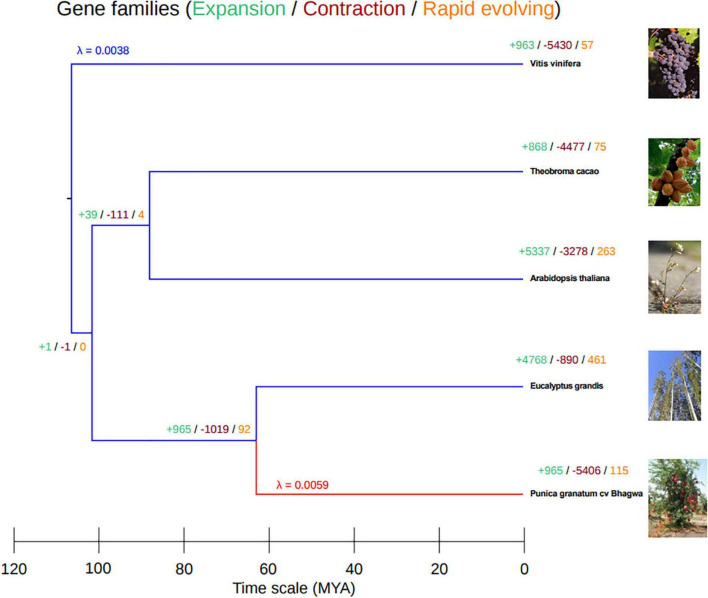
Phylogenetic tree based on gene families by using Computational Analysis of Gene Family Evolution (CAFE) software.

To reconfirm the phylogenetic position of our newly sequenced pomegranate, cv. ‘Bhagawa,’ and to identify expansion, contraction, and rapidly evolving gene families in the *Punica* clade in relation to four other sequenced plant genomes (Supplementary Figure 1), we performed whole-genome phylogenetic analysis. For this, we used CAFE software after pruning the gene families containing a single species. From these analyzes, pomegranate and *E. grandis* diverged 64 (60–70) million years ago (MYA), after the paleotetraploidy event (109 MYA) was identified in the *E. grandis* genome (Figure 7). Therefore, this whole-genome duplication event is shared by pomegranate and *E. grandis* (Myburg et al., 2014; Yuan J. et al., 2018). We also found a higher divergence period between the grape and pomegranate genomes, which suggests that pomegranate and grape did not undergo a recent genome duplication as per results from syntenic block analysis between pomegranate, Eucalyptus, and grape genomes (Myburg et al., 2014; Yuan J. et al., 2018).

To study the gene families that had expanded or contracted in pomegranate, we have estimated and compared gene gain and loss rates (λ) using the two-lambda model for gene families from pomegranate with those of four other representative ancestral species. In total, 965 gene families had expanded in number; 5,406 gene families had contracted in number; and 115 families had rapidly evolved in the pomegranate genome, with λ value 0.0059 as compared with its most recent common ancestor, Eucalyptus (Supplementary Table 2 and Figure 7). However, the grape genome had the most divergent species with gene family evolution (λ 0.0038) as compared with the other genomes.

### Resistance genes and transcription factor identification

Resistance genes in the ‘Bhagawa’ genome were identified by using the protein sequence of predicted genes and the Plant Stress Protein Database (PSPDB). A total of 1,573 protein-coding genes were identified as resistance genes, classified into 32 domains. Among these, Kinase, TM (577); Kinase, LRR, TM (203); LRR, TM (194); and kinase (132) domains increased in number (Table 5). Transcription factors were predicted by using PlantTFDB, with 1,533 proteins identified as transcription factors in ‘Bhagawa.’ These transcription factors are divided into 57 transcription factor families. Genes related to MYB were highest in number, followed by bHLH, ERF, NAC, and C2H2 families (Table 6).

**TABLE 5:** Disease resistance genes identified in ‘Bhagawa’.

Domain	Number of genes	Domain	Number of genes
Kinase, TM	577	CC, Kinase, LRR, TM	10
Kinase, LRR, TM	203	CC, LRR, TM	9
LRR,T M	194	CC, NBS	8
Kinase	132	LRR, NBS, TIR	6
CC, Kinase, TM	57	CC, LRR, NBS, TIR, TM	5
NBS, TM	57	LRR, NBS	5
LRR, NBS, TIR, TM	55	CC, LRR, NBS	3
LRR, NBS, TM	42	CC, LRR	2
CC, NBS, TM	41	CC, TIR	2
CC, LRR, NBS, TM	37	NBS, TIR	2
LRR	36	TM	2
NBS	19	CC	1
NBS, TIR, TM	19	Kinase, LRR	1
TIR	17	Kinase, NBS, TM	1
CC, Kinase	14	LRR, TIR	1
TIR, TM	14	LRR, TIR, TM	1

**TABLE 6:** Transcription factor families and their counts in ‘Bhagawa’.

TF_family	No. of proteins	TF_family	No. of proteins	TF_family	No. of proteins
MYB	157	TCP	23	EIL	6
bHLH	123	FAR1	21	YABBY	6
ERF	123	ARF	19	BBR-BPC	5
NAC	108	AP2	15	CPP	4
C2H2	97	TALE	15	LSD	3
WRKY	66	CO-like	13	RAV	3
bZIP	59	NF-YB	13	HB-PHD	2
MYB_related	56	ZF-HD	13	HRT-like	2
B3	54	GeBP	12	NF-X1	2
M-type_MADS	53	SBP	12	Whirly	2
GRAS	49	ARR-B	11	LFY	1
G2-like	40	GRF	10	S1Fa-like	1
HD-ZIP	40	NF-YC	10	SAP	1
LBD	37	Nin-like	9	STAT	1
C3H	36	WOX	9	VOZ	1
MIKC_MADS	32	DBB	8	E2F/DP	7
Trihelix	31	HB-other	8	SRS	7
Dof	30	NF-YA	8		
GATA	23	BES1	7		
HSF	23	CAMTA	6		

### Microsatellite identification

The masked genome was used to identify simple sequence repeats (SSRs) by using two different tools (MISA and PERF). Since PERF does not identify complex/compound SSRs, we retained outcomes from MISA only. A total length of 2.52 Mb or approximately 0.7% was identified as SSRs in the genome. Then SSRs were divided into three categories according to their length as follows: (1) ≤ 12 bp, (2) 12–19 bp, and (3) ≥ 20 bp. SSRs ≤ 12 bp were higher in number than the other SSR categories, and mononucleotide SSRs were in very high abundance (Table 7).

**TABLE 7:** Identified simple sequent repeats in pomegranate genome.

Type	Number (≤ 12 bp)	Number (12–19 bp)	Number (≥ 20 bp)	Length covered by SSRs	Software
Mono	63,194	16,369	3,055	10,01,124	MISA and PERF
Di	10,771	28,324	1,9387	11,41,770	
Tri	0	8,641	4,820	2,73,408	
Tetra	0	1,695	1,128	64,698	
Penta	0	0	799	22,000	
Hexa	0	0	553	18,185	
Total	73,965	55,029	29,742	25,21,185	

### MicroRNA (miRNA) classification

**FIGURE 8:**
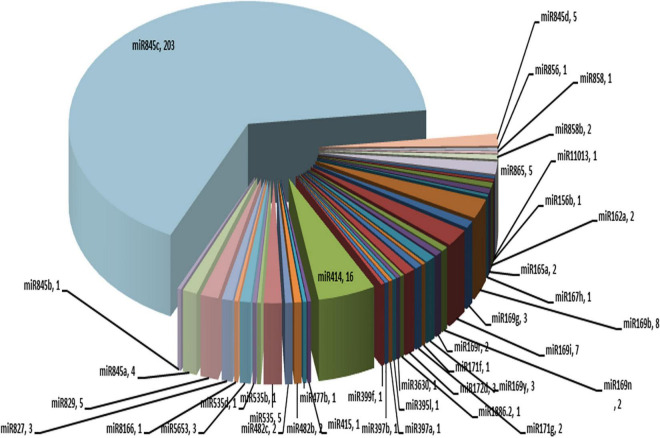
MicroRNAs representing 26 different families identified in the ‘Bhagawa’ genome.

miRNAs are important regulators of several biological processes, such as plant growth and development. These are 20–24 nt in length. A total of 314 copies of miRNAs belonging to 26 different families were identified in the ‘Bhagawa’ genome (Figure 8).

## Discussion

A high-quality complete genome assembly is a valuable genomic resource for identifying structural variants, gene and marker discovery, integrating phenotype–genotype associations, and elucidating the crop’s genome evolution and genetic architecture. The genome sequence information can also be used to understand the phylogenetic position of pomegranate and to identify rapidly evolving gene families in the *Punica* clade in relation to other sequenced plant genomes. The reference-quality pomegranate genome assembled in the present study would certainly be a valuable resource for dissecting many important biological and metabolic traits and a powerful tool to accelerate the pomegranate crop improvement program (Qin et al., 2017).

During the earlier years of the next-generation sequencing revolution, genomes were chiefly assembled by using short-read approaches. However, short-read–based genome assemblies and subsequent data analyzes have major fallacies, such as the inability to span highly repetitive regions longer than the read length and heterozygosity resolution (Xu and Wang, 2007; Simao et al., 2015; Ananthasayanam et al., 2019). The latest fourth-generation sequencing platforms, such as PacBio Sequel, 10X Genomics, and Optical mapping using BioNano, can play a significant role in improving the quality of assembled genomes with a considerable reduction in cost and time required for sequencing. These tools can generate long-read sequences aimed to resolve the above issues but with significantly more errors. So, using a hybrid approach with third-generation sequencing technologies, such as Illumina, and fourth-generation sequencing platforms, such as PacBio Sequel, the quality of the genome assembly can be improved significantly at an affordable cost. The quality of the genome assembled using the hybrid technologies are highly efficient to those assembled using only one sequencing platform. We have demonstrated how a combination of commercially available technologies from Illumina, 10X Genomics, and BioNano Genomics can efficiently be used to assemble high-quality *de novo* sequence scaffolds. Optical mapping for super-scaffolding on long PacBio reads and 10X chromium synthetic-linked reads for assembling a highly contiguous genome was also recommended by other researchers (Mostovoy et al., 2016; Ananthasayanam et al., 2019).

The N50 value of 16.12 Mb, genome coverage of more than 98% within 342 scaffolds, LAI value of 10, and 93.68% BUSCO completeness score over the 1,440 ortholog genes of the complete pomegranate genome indicate the quality of the pomegranate genome assembled in the present study. The better N50 contig length (6.8 Mb) and high N50 scaffold value (16.12 Mb) as compared to the published pomegranate genomes of ‘Taishanhong’ (97 Kb, 1.7 Mb) and ‘Dabenzi’ (82.31 Kb, 2.3 Mb), as well as covering more than 98% of the genome in 342 scaffolds as compared to 2117 and 2601 scaffolds for a lesser percentage of genome coverage in ‘Taishanhong’ and ‘Dabenzi,’ respectively, indicates the high quality of the assembled genome of pomegranate cv. ‘Bhagawa’ (Qin et al., 2017; Yuan Z. et al., 2018). Programs such as the LTR retriever RepeatMasker and RepeatModeler are mostly used for accurate *de novo* identification of intact LTR retrotransposons (Xu and Wang, 2007; Ellinghaus et al., 2008; Ou and Jiang, 2018; Zhang et al., 2019). More intact LTR elements could be identified from more completed genomes as compared with draft genomes (Ou and Jiang, 2018). In turn, the amount of identifiable intact LTR elements can indicate the assembly quality of the intergenic and repetitive sequence space. The LAI can be an indicator of the completeness of the genome, where LAI < 10 can be considered draft assembly, LAI 10–19 reference-quality assembly, and LAI ≥ 20 gold-standard assembly (Ou et al., 2018).

Similarly, completeness is better gaged by using a set of genes that are universally distributed as orthologs across particular clades of species (Treangen and Salzberg, 2012). A summary of complete single-copy, duplicated, fragmented, and missing BUSCO genes is often provided as a quantitative measure of genome completeness based on expected gene content (Simao et al., 2015; Seppey et al., 2019). These transcription factors play a pivotal role in the developmental regulation of gene expression and response of plants to various biotic and abiotic stresses. The most predominant transcription factors in the pomegranate genome were MYB, bHLH, ERF, NAC, and C2H2 families, and these are involved in defense, developmental regulation of gene expression, the response of plants to various biotic and abiotic stresses, and the detoxification response related to drought (Singh et al., 2012; Mittal et al., 2018). The bHLH family is related to drought stress by regulating stomatal development, meristemoid differentiation, and guard cell morphogenesis (Pillitteri et al., 2007). miRNAs are important regulators of several biological processes and stress responses. We identified 314 copies of miRNA belonging to 26 different families (Jones-Rhoades et al., 2006; Sunkar et al., 2007; Singh et al., 2012).

The reference-quality genome assembly of the ‘Bhagawa’ genome in the current study will be an invaluable resource to molecular studies in ‘Bhagawa’ and other species related to pomegranate.

## Materials and methods

### Plant materials

The most commercially grown and popular Indian pomegranate cultivar ‘Bhagawa’ was chosen for genome sequencing. The variety is currently being maintained at field Gene Bank of ICAR-NRCP, Solapur, India. This variety has a medium plant height (1.5 mt), bearing deep red fruits with large size (>250 g), thick rind having dark red-colored bold arils with the soft seed type. The soft red arils of this variety are mainly preferred for edible and processing purposes because of their higher juice percentage (45%), TSS (15.9°Brix), vitamin C (14.60 mg/100 g fresh arils), anthocyanin (360 mg/100 g), iron (0.32 mg/100 g), and zinc (0.50 mg/100 g) content. This variety is a late-maturing type (180 days from flowering to harvesting) with a high yield potential (20 tonnes/ha) widely grown across the country. The newly emerged leaves were taken for DNA isolation and subsequent downstream operations.

### Genome sequencing

High-molecular-weight genomic DNA (>50 Kb) extraction and purification were performed using the Genomic-tip 100/G genomic DNA isolation kit (Qiagen). DNA concentration was measured by using NanoDrop (Thermo Fisher Scientific) and Qubit 2.0 (Invitrogen) instruments. The integrity and quality of genomic DNA were confirmed by using the Bio-Rad^®^ CHEF Mapper^®^ XA Pulsed Field Electrophoresis system. High-quality sequencing data were generated using four different sequencing and mapping technologies (i.e., Illumina, 10X Chromium, PacBio Sequel, and DLS BioNanoSaphyr optical mapping). Illumina sequencing libraries were constructed by using the NEBNext UltraTM DNA Library Prep Kit (Illumina). The 10X Chromium genomic libraries were prepared by using the Chromium Genome HT Library Kit and Gel Bead Kit v2. The SMRTbell library was prepared using SMRTbell Express Template Preparation Kit. For the construction of optical genome maps, standard BioNano protocols, nicking, labeling, repair, and staining processes were implemented. Specifically, DNA was digested by the single-stranded nicking endonuclease Nt.BspQI. Optical maps were assembled with BioNano Irys View analysis software; only single molecules with a minimum length of 100 kb and six labels per molecule were used.

### Genome assembly

We performed a genome survey using the 10X Chromium datasets before the genome assembly. Initially, the data were de-barcoded by using scaff_reads from Scaff10x v4. Kmer-genie was used to generate histograms at multiple k-mers. The histogram from *k* = 31 was used to run GenomeScope v1.0 at a maximum k-mer coverage of 10,000 for estimating the genome size, heterozygosity, and repetitive and unique content of the genome (Vurture et al., 2017). After this, we proceeded with genome assembly step by step by using various datasets obtained from the sequencing. The genome was assembled by using the Falcon and Phased falcon-unzip module (Chin et al., 2017). The unzipped genome was polished by using Arrow as a part of the unzipping pipeline. Then the genome was scaffolded iteratively twice by using Scaff10X v4^3^ with the de-barcoded 10X Chromium reads. BioNano Tools 1v.3.8041.8044 and Solve 3.3_10252018 were used to build a consensus genome map by using molecules > 150 Kb long and hosting 8 labels (Stankova et al., 2016).

The *de novo* assembled Optical Maps were then used to super-scaffold the Scaff10X scaffolded genome by using the Hybrid Scaffolds tool from the same BioNano solve release (Stankova et al., 2016). Gaps introduced by both Scaff10X and Hybrid Scaffolds toolkits were filled by using PBJelly2 once and later by polishing using Pilon, and BUSCO v3 used with Embryophyta lineage constituting 1440 orthologs was performed at each step to determine the completeness of the genome assembly (English et al., 2012; Walker et al., 2014; Simao et al., 2015). Additionally, on the Pilon-polished genome, we mapped transcriptome data (SRR5187757, SRR5187758, SRR5187763, and SRR5187764) of four samples by using HiSAT2 to check for gene-model completion and to aid in the annotation process (Kim et al., 2014; Geib et al., 2018). Deep-Sequenced Illumina short-read datasets were mapped to the genome by using BWA, and subsequent alignments were used to polish the genome by using Pilon. Assemblies from the ‘Dabenzi’ and ‘Taishanhong’ cultivars were aligned to ‘Bhagawa’ by using the “asm5” parameter from minimap2. Various other genomes, *Arabidopsis thaliana, Vitis vinifera, E. grandis*, and *Theobroma cacoa*, were aligned with ‘Bhagawa’ by using the “asm10” parameter. Variants were called from the alignments by using Assemblytics (Nattestad and Schatz, 2016), commit df5361f from GitHub from sizes 1–10,000, with a unique anchor length of 10 kb.

Circos Jupiter graphs were plotted to check the assembly continuity and any misassembles between the ‘Dabenzi’ and ‘Taishanhong’ genomes with respect to the ‘Bhagawa’ genome. We used the largest scaffolds representing 75% of the genome length, consisting of scaffolds > 100 Kb long and with no breakages for at least 50 Kb. Scaffolds were broken into fragments if there was a continuous stretch of 100 Kb, then “N”’s had to be removed (i.e., false alignments). The alignments were computed by using Minimap2 with the parameter “asm10”^4^.

### Genome annotation

To identify and classify different repeats in the pomegranate genome sequences, we used the RepeatModeler-open-1.0.10 pipeline to construct a *de novo* repeat library (Smit et al., 2008). Repeat sequences related to the *Punica* genus were obtained from the RepeatMasker library to classify the repeats by using RepeatClassifier. *De novo* identified repeat sequences, and plant-related repeat sequences were merged to create a custom library. Masking of the genome involved using RepeatMasker v 4.0.9 with the custom library (Tarailo-Graovac and Nansheng, 2009).

We created an orthologs protein set of 4 plant species, including *V. vinifera*, *P. granatum* (‘Dabenzi’), *T. cacao*, and *E. grandis* by using OrthoVenn 2 (Wang et al., 2015). Ortholog protein sets shared by at least two species were submitted to BRAKER2.0 using –prg = gth and –prot_seq options. GTFs from the mapped transcriptome data were merged by using StringTie v1.3.6 to create a catalog of putative transcripts (Pertea et al., 2015). BRAKER2.0 was used to train Augustus v3.3 with the merged GTF and protein alignments (Stanke et al., 2006). Gene models were further improved *via* MAKER2 by providing the gff file generated by using Augustus v3.3, the protein alignment file generated through genome threader (gth), and a CDS file generated by using StringTie v1.3.6; MAKER2 was run iteratively twice to refine the gene predictions (Cantarel et al., 2008).

The predicted gene set was annotated by using EggNOG mapper and blasted against the UniProt^5^ *Viridi plantae* dataset and InterProScan to filter out false-positives generated by MAKER2 (Van Nguyen and Dominique, 2009; Jones et al., 2014; Huerta-Cepas et al., 2017). The curated gene sets were re-annotated by using the same databases. Finally, genome Annotation Generator (GAG) v2.0. was used to add start and stop codons and identify various metrics based on the annotated GFF (Geib et al., 2018). Genes flagged as overlapping or contained by GAG were removed by using BedTools Intersect.

### Microsatellites identification

High-throughput SSRs were identified by using MISA (Beier et al., 2017) and PERF v0.2.5 with the same parameters (Avvaru et al., 2017). The parameters used were minimum SSR motif length of 10 bp and repeat length mono-10, di-6, tri-5, tetra-5, penta-5, and hexa-5; the maximum size of interruption allowed between two different SSRs in a compound sequence was 100 bp. Concordant SSRs between the two approaches were chosen by using Bedtools Intersect (Quinlan, 2010) with a 90% reciprocal overlap.

## Resistance genes and transcription factor identification

Plant stress resistance genes were downloaded from Plant Stress Protein Database (PSPDB^6^ ^,7^) (Kumar et al., 2014). To identify resistance genes in pomegranate, we performed a Blastp of predicted protein sequences of pomegranate against downloaded protein sequences of resistance genes with e-value cutoff (1e-5), query coverage (50%), and sequence identity (50%) (Altschul et al., 1997). Transcription factors were predicted by using PlantTFDB V4.0 by selecting Arabidopsis as a reference (Geib et al., 2018).

### Orthologs identification and estimated gene gain and loss rates

We used Orthofinder 2.3.3 to define gene families across five genomes: *P. granatum* cv. ‘Bhagawa,’ *A. thaliana, E. grandis, T. cacao*, and *V. vinifera* downloaded from NCBI(Emms David and Steven, 2015). We used the options Diamond to blast, MSA using MAFFT, and tree inference using raxml-ng (Katoh et al., 2005; Buchfink et al., 2015). CAFE 4.2.1 was used to identify expansion, contraction, and rapidly evolving gene families in the *Punica* clade (De Bie et al., 2006; UniProt Consortium, 2014; Kozlov et al., 2019; Singh et al., 2021). We estimated gene gain and loss rates with a two-lambda model.

## Data availability statement

The original contributions presented in this study are publicly available. The NCBI data accession numbers: PRJNA445950, PRJNA562100, PRJNA505392, PRJNA505397, PRJNA505398, and PRJNA505582.

## Author contributions

PR, PS, NS, and PP: conceptualization of idea, planning of research experiments, collection and processing of samples, compilation of data, secondary analysis of data, and drafting the manuscript. PN and UR: compilation of data, and secondary analysis of data and drafting the manuscript. JS, KB, and RM: drafting the manuscript and review of the manuscript. VS and DM: collection and processing of samples. All authors contributed to the article and approved the submitted version.

## Footnotes

1
http://nhb.gov.in

2
http://agriexchange.apeda.gov.in

3
https://github.com/wtsi-hpag/Scaff10X

4
https://github.com/JustinChu/JupiterPlot.git

5
www.uniprot.org

6
https://bioclues.org

7
https://www.ncbi.nlm.nih.gov/
