# Supplementary material for: Reference quality genome sequence of Indian pomegranate cv. ‘Bhagawa’ (Punica granatum L.)
Source: Front Plant Sci. 2022 Sep 15;13:947164. doi: 10.3389/fpls.2022.947164 (PMC9521485; doi:10.3389/fpls.2022.947164)
Supplement: Supplementary file 1 [file Data_Sheet_1.docx]

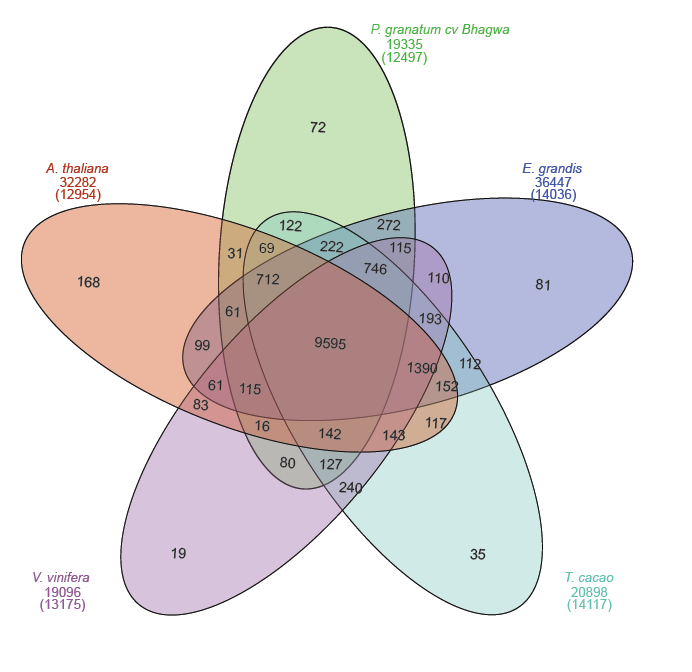


**Supplementary Figure 1:** Asymmetric venn diagram showing the Ortholog gene-families as identified by Orthofinder.

**Supplementary Table 1a: Counts of identified variants**

|  |  | **Taishanhong** | **Dabenzi** | **Grape** | **Eucalyptus** |
| --- | --- | --- | --- | --- | --- |
|  | **Parameter** | **Asm5** | **Asm5** | **Asm10** | **Asm10** |
| **Insertion** | **Size range** |  |  |  |  |
|  | 1-10 bp | 28181 | 29031 | 104 | 172 |
|  | 10-50 bp | 3922 | 8352 | 19 | 21 |
|  | 50-500 bp | 1819 | 9683 | 2 | 1 |
|  | 500-10000 bp | 251 | 1047 | 2 | 1 |
| **Total** |  | **34180** | **48125** | **127** | **195** |
|  |  |  |  |  |  |
| **Deletion** | **Size range** |  |  |  |  |
|  | 1-10 bp | 29931 | 29278 | 108 | 311 |
|  | 10-50 bp | 4608 | 4189 | 11 | 15 |
|  | 50-500 bp | 1658 | 2762 | 1 | 3 |
|  | 500-10000 bp | 378 | 658 | 0 | 0 |
| **Total** |  | **36608** | **36905** | **120** | **329** |
|  |  |  |  |  |  |
| **Tandem_expansion** | **Size range** |  |  |  |  |
|  | 1-10 bp | 1 | 4 | 0 | 0 |
|  | 10-50 bp | 1 | 0 | 0 | 0 |
|  | 50-500 bp | 3 | 36 | 0 | 0 |
|  | 500-10000 bp | 198 | 884 | 0 | 0 |
| **Total** |  | **203** | **924** | **0** | **0** |
|  |  |  |  |  |  |
| **Tandem_contraction** |  |  |  |  |  |
|  | 1-10 bp | 0 | 1 | 0 | 0 |
|  | 10-50 bp | 0 | 1 | 0 | 0 |
|  | 50-500 bp | 2 | 4 | 0 | 0 |
|  | 500-10000 bp | 163 | 40 | 0 | 0 |
| **Total** |  | **165** | **46** | **0** | **0** |
|  |  |  |  |  |  |
| **Repeat_expansion** |  |  |  |  |  |
|  | 1-10 bp | 170 | 391 | 3 | 1 |
|  | 10-50 bp | 201 | 769 | 4 | 5 |
|  | 50-500 bp | 290 | 1599 | 4 | 4 |
|  | 500-10000 bp | 145 | 273 | 1 | 1 |
| **Total** |  | **806** | **3032** | **12** | **11** |
|  |  |  |  |  |  |
| **Repeat_contraction** | **Size range** |  |  |  |  |
|  | 1-10 bp | 125 | 271 | 0 | 1 |
|  | 10-50 bp | 242 | 323 | 4 | 2 |
|  | 50-500 bp | 272 | 1064 | 6 | 2 |
|  | 500-10000 bp | 188 | 852 | 0 | 3 |
|  |  |  |  |  |  |
| **Total** |  | 827 | 2510 | 10 | 8 |
| **Total for all variants** |  | 72789 | 91542 | 269 | 543 |
| **Total for all structural variants** |  | 5407 | 18932 | 16 | 15 |

**Supplementary Table 1b: Lengths of identified variants**

|  |  | **Taishanhong** | **Dabenzi** | **Grape** | **Eucalyptus** |
| --- | --- | --- | --- | --- | --- |
|  | **Parameter** | **Asm5** | **Asm5** | **Asm10** | **Asm10** |
| **Insertion** | **Size range** | **Total bp** | **Total bp** | **Total bp** | **Total bp** |
|  | 1-10 bp | 61184 | 65046 | 380 | 606 |
|  | 10-50 bp | 82348 | 207897 | 327 | 334 |
|  | 50-500 bp | 228836 | 1474885 | 466 | 390 |
|  | 500-10000 bp | 665276 | 1279317 | 1260 | 664 |
| **Total (bp)** |  | **1124172** | **3177873** | **2433** | **1994** |
|  |  |  |  |  |  |
| **Deletion** | **Size range** |  |  |  |  |
|  | 1-10 bp | 65983 | 63099 | 418 | 672 |
|  | 10-50 bp | 92337 | 86300 | 174 | 242 |
|  | 50-500 bp | 246339 | 473537 | 105 | 199 |
|  | 500-10000 bp | 1083436 | 1206886 | 0 | 0 |
| **Total (bp)** |  | 1961625 | 2089273 | 697 | 1113 |
|  |  |  |  |  |  |
| **Tandem_expansion** | **Size range** |  |  |  |  |
|  | 1-10 bp | 1 | 6 | 0 | 0 |
|  | 10-50 bp | 34 | 0 | 0 | 0 |
|  | 50-500 bp | 733 | 8672 | 0 | 0 |
|  | 500-10000 bp | 773789 | 1439921 | 0 | 0 |
| **Total (bp)** |  | **774557** | **1448599** | **0** | **0** |
|  |  |  |  |  |  |
| **Tandem_contraction** | **Size range** |  |  |  |  |
|  | 1-10 bp | 0 | 2 | 0 | 0 |
|  | 10-50 bp | 0 | 15 | 0 | 0 |
|  | 50-500 bp | 523 | 1292 | 0 | 0 |
|  | 500-10000 bp | 584318 | 120623 | 0 | 0 |
| **Total (bp)** |  | **584841** | **121932** | **0** | **0** |
|  |  |  |  |  |  |
| **Repeat_expansion** | Size range |  |  |  |  |
|  | 1-10 bp | 707 | 1639 | 14 | 8 |
|  | 10-50 bp | 4982 | 20233 | 98 | 197 |
|  | 50-500 bp | 57554 | 291579 | 849 | 795 |
|  | 500-10000 bp | 334384 | 508330 | 748 | 1179 |
| **Total (bp)** |  | **397627** | **821781** | **1709** | **2179** |
|  |  |  |  |  |  |
| **Repeat_contraction** | **Size range** |  |  |  |  |
|  | 1-10 bp | 453 | 958 | 0 | 4 |
|  | 10-50 bp | 6687 | 8294 | 119 | 43 |
|  | 50-500 bp | 41585 | 229056 | 1120 | 190 |
|  | 500-10000 bp | 584248 | 1337616 | 0 | 8809 |
| **Total (bp)** |  | **632973** | **1575924** | **1239** | **9046** |
|  |  |  |  |  |  |
| **Total for all variants (bp)** |  | **5475795** | **9235382** | **6078** | **14332** |
| **Total for all structural variants (bp)** |  | **5161079** | **8781893** | **4548** | **12226** |


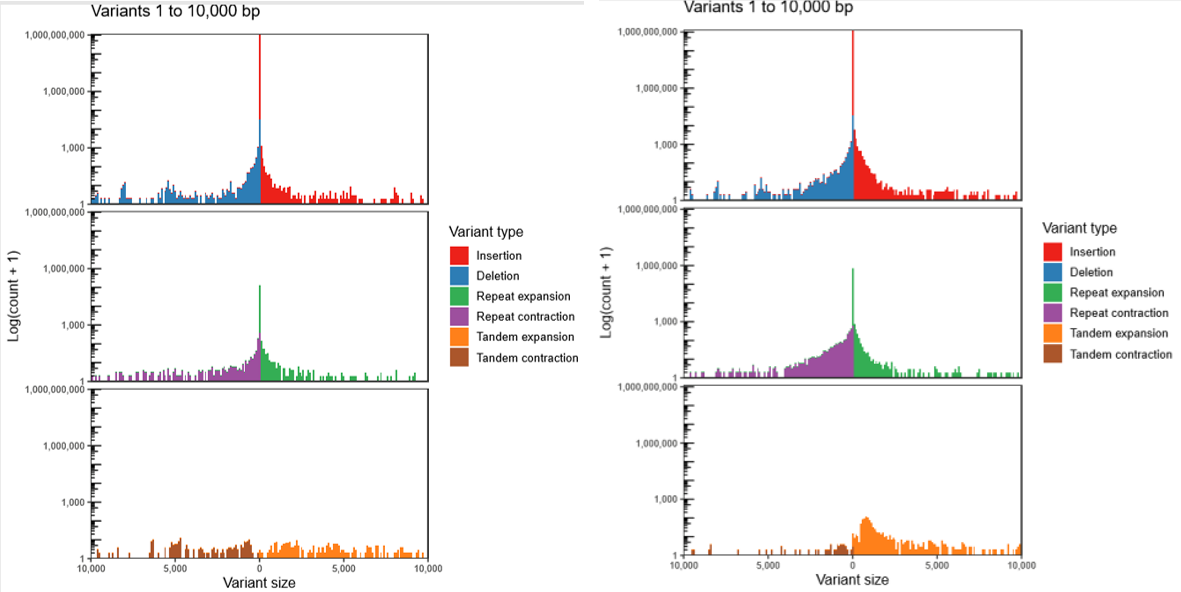


**b. Bhagawa Vs. Dabenzi**

1. **Bhagawa Vs. Taishanhong**

**SUPPLEMENTARY FIGURE 2.** Structural variation analysis of ‘Bhagawa’ *vis-a-vis* (i) Taishanhong (ii) Dabenzi


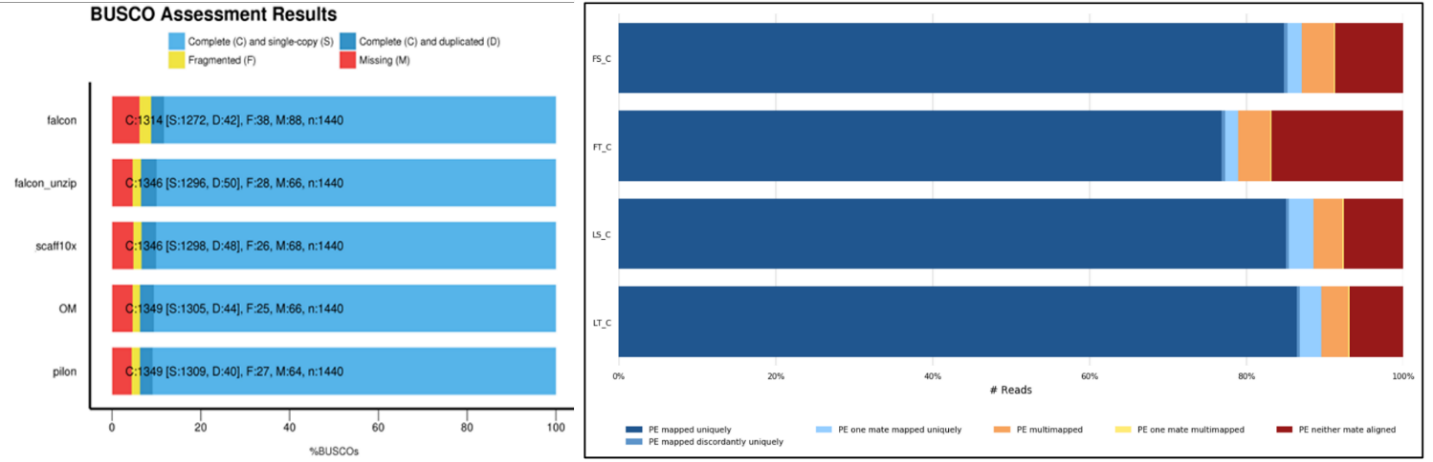


**SUPPLEMENTARY FIGURE 3:** Genome completeness and genome mapping analysis to validate the quality of genome assembly from ‘Bhagawa’

**SUPPLEMENTARY TABLE 2:** Orthogene families shared by all the five species

| **Number of species** | 5 |
| --- | --- |
| **Number of genes** | 168848 |
| **Number of genes in orthogroups** | 119942 |
| **Number of unassigned genes** | 48906 |
| **Percentage of genes in orthogroups** | 71 |
| **Number of orthogroups** | 18973 |
| **Number of species-specific orthogroups** | 346 |
| **Number of genes in species-specific orthogroups** | 195 |
| **Percentage of genes in species-specific orthogroups** | 1.2 |
| **Mean orthogroup size** | 6.3 |
| **Median orthogroup size** | 5 |
| **Number of orthogroups with all species present** | 9815 |
| **Number of single-copy orthogroups** | 3710 |
